# Flower and Leaf Extracts of *Sambucus nigra* L.: Application of Membrane Processes to Obtain Fractions with Antioxidant and Antityrosinase Properties

**DOI:** 10.3390/membranes9100127

**Published:** 2019-09-24

**Authors:** Rosa Tundis, Claudia Ursino, Marco Bonesi, Monica R. Loizzo, Vincenzo Sicari, Teresa Pellicanò, Ilaria L. Manfredi, Alberto Figoli, Alfredo Cassano

**Affiliations:** 1 Department of Pharmacy, Health and Nutritional Sciences, University of Calabria, I-87036 Rende (Cosenza), Italy; rosa.tundis@unical.it (R.T.); marco.bonesi@unical.it (M.B.); 2 Institute on Membrane Technology, Italian National Research Council, ITM-CNR, I-87036 Rende (Cosenza), Italy; c.ursino@itm.cnr.it (C.U.); ilariamanfredi89@gmail.com (I.L.M.); a.cassano@itm.cnr.it (A.C.); 3 Department of Agricultural Science, Mediterranean University of Reggio Calabria, I-89123 Reggio Calabria, Italy; vincenzo.sicari@unirc.it (V.S.); teresa.pellicano@unirc.it (T.P.)

**Keywords:** *Sambucus nigra*, flowers, leaves, tyrosinase inhibition, antioxidant activity, OSN, ECTFE

## Abstract

This study aimed at evaluating and comparing the chemical profile as well as the antityrosinase and antioxidant activities of ethanol (EtOH) and methanol (MeOH) extracts of *Sambucus nigra* L. (Adoxaceae) flowers and leaves in order to discover new candidates for food additives and cosmetic and pharmaceutical products. For this purpose, a novel lower-melting-point ethylene-chlorotrifluoroethylene (LMP ECTFE) nanofiltration (NF) membrane was employed in order to produce the concentrated fractions of *S. nigra*. Floral extracts were richer in phytochemicals in comparison to the leaf extracts. The High-performance liquid chromatography (HPLC) profile revealed rutin, quercetin, protocateuchic acid, 3,5-dicaffeoylquinic acid, and neochlorogenic acid as the most abundant compounds. Ferric reducing antioxidant power (FRAP), 2,2’-diphenil-1-picrylhydrazil (DPPH) radical scavenging, and 2,2’-azino-bis-(3-ethylbenzothiazoline-6-sulfonic acid) (ABTS) tests were used to investigate the antioxidant properties. NF retentate fractions of floral ethanol extracts exerted the highest tyrosinase inhibitory activity with an IC_50_ of 53.9 µg/mL and the highest ABTS radical scavenging activity (IC_50_ of 46.4 µg/mL). In conclusion, the present investigation revealed the potential benefits of NF application in *S. nigra* extracts processing, suggesting the use of retentate fractions as a promising source for antioxidant and tyrosinase inhibitory compounds which could pave the way for future applications.

## 1. Introduction

*Sambucus nigra* L. (elder) is a well-known species of the Caprifoliaceae family common in Europe, North Africa, USA, and West Asia. Its flowers are used to flavor wine and to make tea and nonalcoholic cordial. Moreover, they are added to the batter that is used for the preparation of foodstuffs such as waffles, pancakes, and muffins [1]. *S. nigra* was reported to possess several traditional medicinal properties including antidiabetic, purgative, diuretic, hemostatic, and diaphoretic properties [2,3].

The biological properties of *S. nigra* are closely related to its chemical constituents. Anthocyanins, other polyphenolic compounds, and organic acids, known for their antioxidant potential activity, are the dominant classes of phytochemicals that characterize *S. nigra*. 

The recovery of natural antioxidants from plant extracts, especially phenols and flavonoids, has gained a growing interest in the last decades in view of the accepted use of these compounds as food additives or nutritional supplements [4,5]. When exposed to ultraviolet (UV) radiation, the human skin produces reactive oxygen species (ROS) that in turn are able to activate different biological responses. Mounting ROS levels activate tyrosinase by mobilizing α-melanocyte-stimulating hormone in the epidermis and finally stimulates the melanocytes to produce melanin. Meanwhile, the Keap1-Nrf2/ARE pathway, which removes ROS, is activated at increased ROS levels, and antioxidant compounds facilitate the dissociation of Nrf2. Tyrosinase is the key enzyme during the melanogenesis [6].

The development of integrated processing systems able to minimize the degradation of bioactive compounds throughout raw material handling, processing, packaging, and storage of the final supplement is a key objective in nutraceuticals processing. In this view, membrane processes have gained a growing interest in the last years due to their typical advantages over conventional separation methodologies, including mild operating conditions of temperature and pressure, non-use of chemical agents or solvents, and reduced waste treatment volume and costs [7,8].

In our previous work, the chemical profile, physico-chemical parameters, and antioxidant and hypoglycemic properties of *S. nigra* juice, treated with commercial nanofiltration (NF) membranes of different polymeric material (composite fluoropolymer and polyethersulphone) and molecular weight cut-off (400 and 1000 Da), were analyzed [9]. Experimental results indicated that most parts of the bioactive compounds were retained by the NF membranes producing a retentate fraction of interest for the production of functional foods.

This study was aimed at investigating the potential suppressing effects of antioxidant and tyrosine inhibitory compounds identified in flower and leaf extracts of *S. nigra*. In addition, the use of organic solvent nanofiltration (OSN) was explored to concentrate these compounds from both extracts. OSN, also known as solvent-resistant nanofiltration (SRNF), is an emerging technology for the separation of molecules with molecular weights between 200 and 2000 g·mol^−1^ in various organic solvents by employing a pressure gradient across a membrane [10]. Generally, it is said that the pore size range of the membrane falls between 1 and 10 nm [11]. OSN offers several advantages over conventional energy-intensive processes like distillation, evaporation, and liquid–liquid extraction as no phase transitions are involved, thus lowering remarkably the energy consumption. Additional advantages include milder operating temperatures, thus preventing thermal degradation of thermo-sensitive compounds, lower operating costs, and the possibility to recycle solvents, thus reducing the production of waste and depollution costs [12,13]. Polymeric materials are widely used for membrane preparation as they offer a great variety of different choices of polymers, cost-effectiveness, excellent processability, and good reproducibility. However, full-scale applications of OSN are still limited because most available commercial NF membranes present limited solvent (chemical) and thermal stabilities. Therefore, the choice of the polymeric material represents one of the crucial elements to predict performance and stability of the OSN process [14,15]. Polymeric materials commonly used in the preparation of OSN membranes present some limitations; for example, the polysulfone family is less resistant to oxidation agents and can be attacked by hydrophobic solvents and plasticizers [16], polyamide membranes are not resistant to acids and present limited or high resistance to bases [17,18], while polyvinylidene fluoride membranes are sensitive to caustic solutions and bases as amines [19]. Today, the research pays attention to new materials able to give mechanical, chemical, and thermal stability and at the same time are easily workable [20]. Recently, the use of fluoropolymers in different membrane processes has become popular. The C–F chemical bond is the strongest known single bond in organic chemistry leading to a great stability of these polymers. In addition, polymers containing C–F bonds exhibit an excellent chemical resistance, high thermal stability, reduced adhesion to surfaces, high mechanical strength, and low surface tension [21]. As a result of this stability, often many fluoropolymers are difficult to work with. In the present work, a novel class of NF membranes, produced by using a suitable solvent-resistant polymer, named lower-melting-point ethylene-chlorotrifluoroethylene (LMP ECTFE), was employed. This polymer developed by Solvay Specialty Polymers (Bollate, Mi) is a lower-melting-point grade of standard Halar^®^ ECTFE, but is easily workable [22]. As reported in literature, ECTFE membranes were successfully applied in the pervaporation (PV )process for aqueous solutions of toluene (200–250 ppm), with an enrichment factor (β) of 894 and 36 g m^-2^ h^-1^ of toluene flux [23]. Falbo et al. reported the use of ECTFE PV membranes for binary azeotropic mixture of ethanol and cyclohexane (30.5% *w/w* and 69.5% *w/w*) obtaining flux and selectivity of 1.7 kg m^2^h and 15, respectively [24]. ECTFE flat membranes were also employed in a membrane condenser by Drioli et al. [25], with a water recovery between 35% and 55%. Moreover, ECTFE porous membranes fabricated using a ternary system showed excellent fouling resistance during the vacuum membrane distillation process (VMD), with a salt rejection (3.5 wt% NaCl) exceeding 99.99% [26]. Thanks to the properties of LMP ECTFE polymer, such as excellent chemical and mechanical resistance, these novel membranes result in a higher stability toward organic solvents and they are suitable for extraction processes based on the use of alcohols [22,27].

## 2. Materials and Methods

### 2.1. Chemicals and Reagents 

Ascorbic acid, butylated hydroxytoluene (BHT), kojic acid, L-tyrosine, mushroom tyrosinase, sodium molybdate, sodium nitrite, sodium hydroxide, sodium carbonate, 2,2′-azino-bis(3-ethylbenzothiazoline-6-sulphonic acid) (ABTS), 2,2-diphenyl-1picrylhydrazyl (DPPH), ethanol (EtOH), and methanol (MeOH) were purchased from Sigma-Aldrich (Milan, Italy). Analytical-grade solvents and all HPLC-grade solvents were obtained from Carlo Erba Reagents (Milan, Italy). LMP ECTFE polymer was supplied by Solvay Specialty Polymers (Bollate, MI). Standards for HPLC analyses were purchased by Extrasynthese (Genay-France).

### 2.2. Plant Materials and Extraction Procedure 

*S. nigra* flowers and leaves were collected in Southern Italy (Calabria) in May 2015 in Tarsia (Cosenza, Italy) (Latitude 39°37’18”84 N, Longitude 16°16’21”00 E) and identified by Dr. N.G. Passalacqua, Natural History Museum of Calabria and Botanic Garden, University of Calabria. Fresh flowers and leaves were extracted by maceration using ethanol and methanol (3 × 72 h) as solvents. The extractive solutions were collected, the solvent was evaporated by using a rotary evaporator at 35 °C to dryness, and the residue was dried under vacuum. Yields of 6.7 and 5.0% were obtained for the extraction of flowers by ethanol and methanol, respectively. Yields of 7.2 and 7.6% were obtained for the extraction of leaves by ethanol and methanol, respectively.

### 2.3. Nanofiltration Set-Up 

LMP ECTFE NF membranes used in this work, named N2, were prepared and characterized as reported in detail by Ursino et al. [22]. Membrane properties are summarized in Table 1.

**Figure 1:**
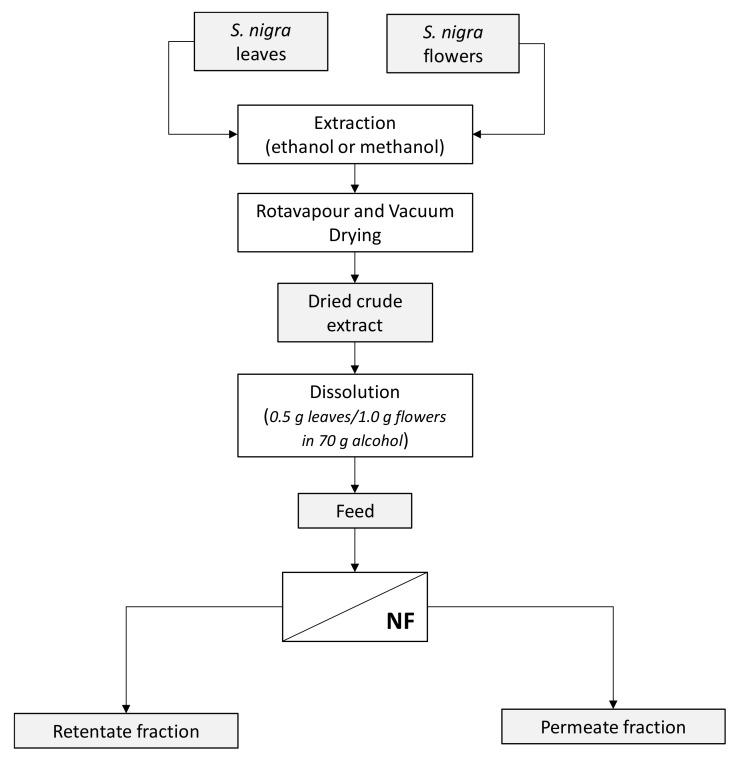
Extraction of *S. nigra* leaves and flowers and concentration of bioactive compounds by nanofiltration.

Filtration tests were performed by using a high-pressure crossflow filtration cell (model HP4750) supplied by Sterlitech Corporation. The cell presented a volume of 300 mL and a diameter of 5.1 cm, with an effective membrane area of 20.4 cm^2^. The membrane was preventively conditioned by immersion for 24 h in the target pure solvent (EtOH or MeOH) before the experiments. After conditioning, the membrane was placed in the cell. The permeate was collected by applying an N_2_ gas pressure (transmembrane pressure) at 12 bar and room temperature. Each test was conducted three times. The permeate flux (*J*) was calculated by the following equation:

(1) $$J=\frac{V}{A\cdot \Delta t}$$

where *V* (L) is the volume of permeate, *A* (m^2^) is the membrane area, and Δ*t* (h) is the operation time. The average and relative standard deviations were calculated. A schematic of the extraction and filtration procedure of *S. nigra* samples is illustrated in Figure 1.

### 2.4. RP-HPLC/DAD Analysis

An aliquot of the extract was diluted with MeOH/H_2_O (80:20 *v*/*v*) and before HPLC/DAD analysis, samples were filtered through a 0.45 μm membrane filter (GMF, Whatman). Analyses were performed on a Knauer (Asi Advanced Scientific Instruments, Berlin) system equipped with two pumps Smartiline Pump 1000, a Rheodyne injection valve (20 μL), and a photodiode array detector equipped with a semi micro-cell. The reverse-phase chromatographic separations were carried out on a TSKgel ODS-100V (250 mm × 3.0 mm, 3 μm) at 30 °C. The mobile phase consisted of a solvent A (water/formic acid, 99.9:0.1 *v/v*) and solvent B (acetonitrile/formic acid 99.9:0.1 *v/v*) gradient at flow rate of 0.4 mL/min. The gradient was 0.01–20.00 min 5% B isocratic; 20.01–50.00 min, 5–40% B; 50.01–55.00 min, 40–95% B; 55.01–60.00 min 95% B isocratic. The injection volume was 20 μL. Peaks were monitored at λ = 254, 330, and 305 nm.

The identification and quantification of the main phytochemicals that characterize *S. nigra* flower and leaf extracts were carried out from the retention times in comparison with authentic standards (astragalin, caffeic acid, chlorogenic acid, 3,5-dicaffeoylquinic acid, ferulic acid, isoquercetin, kaempferol, myricetin, neochlorogenic acid, *p*-coumaric acid, protocateuchic acid, quercetin, rosmarinic acid, and rutin). Data processing was carried out using *Clarity* Software (Chromatography Station for Windows). All analyses were performed in triplicate and results were expressed as mg/100 g of extract.

### 2.5. Ferric Reducing Antioxidant Power (FRAP) Test 

This assay is based on the redox reaction that involves TPTZ (2,4,6-tripyridyl-*s*-triazine)-Fe^3+^ complex. The FRAP reagent was freshly prepared by mixing 25 mL of acetate buffer 0.3 M (pH 3.6), 2.5 mL of TPTZ solution 10 mM in HCl 40 mM, and 2.5 mL of FeCl_3_ 20 mM. A concentration of 2.5 mg/mL of *S. nigra* extracts was prepared and 0.2 mL of this solution was mixed with 1.8 mL of FRAP reagent. The reduction of TPTZ-Fe^3+^ complex to TPTZ-Fe^2+^ complex was monitored at 595 nm. This change is directly related to the reducing power of electron-donating antioxidants present in the reaction mixture [28]. BHT was used as positive control.

### 2.6. DPPH Assay 

The DPPH radical scavenging activity of *S. nigra* extracts was assessed by using DPPH assay as previously reported [28]. Reacting with an antioxidant, DPPH radicals were reduced and generated a change in color, read at 517 nm (Perkin Elmer Lambda 40 UV/VIS spectrophotometer, Milan, Italy). 

The DPPH radical scavenging activity was calculated as a percentage of DPPH discoloration using the following equation:

(2) $$\mathrm{DPPH}\;\mathrm{radical}\;\mathrm{scavenging},\;\%=\left(\frac{A_{0}-A}{A_{0}}\right)\cdot 100$$

where *A*_0_ is the absorbance of the DPPH solution and *A* is the absorbance of the sample. Ascorbic acid was used as positive control.

### 2.7. ABTS Assay 

In this assay, the ABTS solution was prepared by the reaction of ABTS 7 mM and potassium persulphate 2.45 mM, and stored (for 12 h) at room temperature in the dark. The solution was diluted with ethanol to reach an absorbance of 0.70 at 734 nm. Then, 25 mL of different concentrations of *S. nigra* extracts were added to 2 mL of diluted ABTS solution and the absorbance was measured at 734 nm after 6 min. The ABTS radical scavenging ability of *S. nigra* extracts was calculated according to the following equation:

(3) $$\mathrm{ABTS}\;\mathrm{radical}\;\mathrm{scavenging},\;\%=\left(\frac{A^{\prime }-A}{A^{\prime }}\right)\cdot 100$$

where *A^’^* is the absorbance of the control reaction and *A* is the absorbance in the presence of the sample [28]. Ascorbic acid was employed as positive control.

### 2.8. Relative Antioxidant Capacity Index (RACI) 

RACI is a statistical index that provides a rank of antioxidant ability generated from different in vitro tests applied to the investigated samples [29]. Herein, data obtained from FRAP, ABTS, and DPPH tests were used to calculate a RACI value for *S. nigra* extracts.

### 2.9. Tyrosinase Inhibitory Activity Test 

The mushroom tyrosinase inhibition assay was performed as previously described [30]. Briefly, 40 μL of mushroom tyrosinase solution (100 units/mL), 40 μL of L-tyrosine solution (0.1 mg/mL), 40 μL of *S. nigra* extract in hydroalcoholic solution, and 80 μL of phosphate-buffered saline (PBS) solution (25 mM, pH 6.8) were added to a 96-well microplate and incubated for 30 min at 37 °C. The amount of the produced dopachrome was measured at 492 nm before and after incubation. Kojic acid was used as positive control. The percentage of tyrosinase inhibition was calculated as follows:

(4) $$\mathrm{Inhibition}\;\left(\%\right)=\left[\frac{\left(A-A^{\prime }\right)-\left(B-B^{\prime }\right)}{\left(A-A^{\prime }\right)}\right]\cdot 100$$

where *A* and *A*^’^ are the absorbance values of the blank solution after and before incubation, respectively; *B* and *B*^’^ are the absorbance values of the extract solution after and before incubation, respectively.

### 2.10. Statistical Analysis 

The half-maximal inhibitory concentration (IC_50_) was calculated by nonlinear regression with the use of GraphPad Prism version 6 for MS Windows (GraphPad Software, San Diego, CA, USA). The concentration–response curve was obtained by plotting the percentage inhibition vs concentration. The results, expressed as the mean values and standard deviations (SD), were analyzed by using the one-way ANOVA test and multicomparison Dunnett’s test.

## 3. Results and Discussion

### 3.1. Evaluation of Membrane Productivity

**Figure 2:**
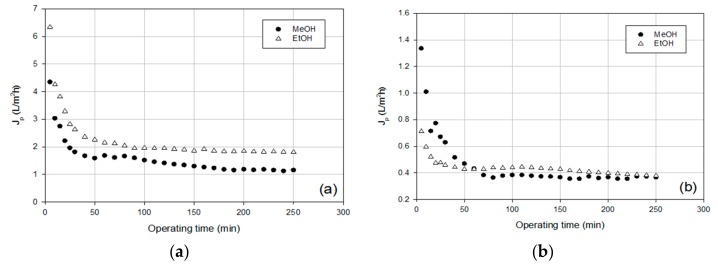
Permeate flux as a function of operating time in nanofiltration of methanol and ethanol extract of *S. nigra* (**a**) leaves and (**b**) flowers.

LMP ECTFE N2 NF membrane was selected based on its properties and performance, as reported in Ursino et al. [22]. Indeed, LMP ECTFE N2 membrane presented a good mechanical performance and excellent chemical stability, with a low degree of swelling in different solvents, in particular, 4% and 6% for methanol and ethanol, respectively. The ethanol and methanol permeability at room temperature were of 3.0 and 3.6 L/m^2^ h bar, respectively. These differences can be attributed to the molecular weight and the viscosity of both solvents [13,22]. The time evolution of permeate flux in the selected operating conditions for both leaf and flower extracts is reported in Figure 2.

The J vs time curve of all extracts showed a typical trend characterized by a rapid initial drop of the permeate flux in the first 10–15 min of filtration, followed by a gradual flux decrease, and ending with a steady-state flux. The initial drop of the permeate flux can be attributed to quick blocking of membrane pores due to retained particles. Further flux decline after pore blockage was due to the formation and growth of a cake layer on the membrane surface which created an additional resistance to the permeate flow. 

The permeate flux of the ethanol extract of *S. nigra* leaves resulted in being higher in comparison to that of the methanol extract with a stationary flux of about 2 L/m^2^ h (Figure 2a). On the other hand, the permeate flux of *S. nigra* flowers in EtOH and MeOH showed a similar trend, with a stationary flux of around 0.4 L/m^2^ h (Figure 2b). Therefore, the membrane productivity with floral extracts was lower than that observed for leaf extracts. This trend can be attributed to the lower solubility of the leaf extracts; indeed, the total concentration of leaf and floral extracts was 0.006 mg/mL and 0.011 mg/mL, respectively. In addition, both floral extracts presented a higher quantity of particulate material resulting in higher turbidity in comparison with the leaf extracts.

### 3.2. Chemical Profile 

*S. nigra* flowers and leaves were extracted by maceration using methanol and ethanol as solvents. The content of selected phenolic acids and flavonoids analyzed in crude extracts and retentate fractions of *S. nigra* flowers and leaves by HPLC-DAD is reported in Table 2 and Table 3, respectively.

Overall, obtained data showed that target compounds exhibited higher content in methanol extracts compared to ethanol extracts. Many techniques, including maceration, subcritical water extraction, Soxhlet extraction, and ultrasound-assisted extraction, are used to recover antioxidants from plants. However, the extraction yield and the antioxidant effects depend not only on the employed extraction method but also on the solvent used for the extraction process. To recover polyphenols from plants, polar solvents are often used. Among these, ethanol has been known as a good solvent in addition to being safe for human consumption. Generally, methanol has been found to be more efficient in extraction of polyphenols with lower molecular weight [31,32]. Moreover, in agreement with previous studies, the flavonoid amounts are greater in flowers than in leaves [33,34,35,36].

Floral extracts were mainly characterized by the presence of rutin, protocateuchic acid, chlorogenic acid, neochlorogenic acid, kaempferol, and quercetin. As expected, with the exception of chlorogenic acid and neochlorogenic acid, retentate fractions of both methanol and ethanol extracts exhibited a higher content of target compounds in comparison with crude extracts. In addition, in the retentate fractions, caffeic acid and *p*-coumaric acid were not detected. These differences with crude extracts can be attributed to the permeation of such compounds through the NF membrane. According to the data in Table 2, concentration of floral extracts by NF increased the content of most bioactive compounds of both alcoholic extracts, including 3,5-dicaffeoylquinic acid, isoquercetin, kaempferol, quercetin, and rosmarinic acid by 120–180%. 

Quercetin, neochlorogenic acid, isoquercetrin, chlorogenic acid, and kaempferol are the most abundant compounds in leaf extracts. However, the content of these constituents is lower than that found in the floral extracts. In addition, ferulic acid, protocateuchic acid, and rosmarinic acid were not identified. As for floral extracts, caffeic acid and *p*-coumaric acid were not detected in the retentate fractions of both methanol and ethanol extracts. Moreover, some target compounds, including chlorogenic acid, 3,5-dicaffeoylquinic acid, and isoquercetin, were more expressed in the crude extracts due to their permeation through the NF membrane (Table 3). On the other hand, the content of quercetin in both alcoholic extracts increased by 225%.

### 3.3. Antioxidant Activity 

The antioxidant properties of *S. nigra* leaf and floral extracts and related NF retentate fractions were estimated by using two radical scavenging assays (ABTS and DPPH tests) and ferric reducing power (FRAP) test. Data are reported in Table 4. All samples showed a concentration-dependent activity.

As expected, both retentate fractions exhibited higher DPPH and ABTS radical scavenging activity than crude extracts. In particular, both leaf and flower retentate fractions of methanol extracts showed the highest activity with IC_50_ values of 39.2 and 48.3 μg/mL, respectively, in DPPH assay. A similar trend was observed in the ABTS test where flower retentate fraction of methanol extract exhibited the highest potency with an IC_50_ value of 46.4 μg/mL. Many chemical reactions including radicals involved iron for its ability to transfer single electrons, starting even with relatively nonreactive radicals [37]. Thus, the reduction of ferric ion is an important approach to investigate the antioxidant potential of samples. In this study, all investigated samples showed higher FRAP values than that reported for BHT used as positive control. In particular, the retentate fraction of leaf ethanol extract exhibited a FRAP value of 110.9 μM Fe(II)/g, which is 1.75 times higher than BHT. A comparable FRAP value was observed also in the retentate fraction of floral methanol extract. Pearson’s correlation coefficient calculated between the sum of quantified compounds and the result of the FRAP assay evidenced an *r* value of 0.64. The integrated approach RACI was used to estimate the sample with the highest antioxidant potential. As reported in Table 4, both crude extract and retentate fraction of leaf methanol extracts showed promising RACI values of −0.52 and −0.51, respectively. The retentate of the floral methanol extract showed the highest RACI value (−0.01) for this set of samples.

Recently, Viapiana and Wesolowski [38] reported that infusion prepared from *S. nigra* flowers had higher mean DPPH and FRAP activities than the teas prepared from berries. The potential ABTS radical scavenging activity confirmed results obtained by Młynarczyk and Walkowiak-Tomczak [39] that demonstrated the methanol extract of fresh flowers was more potent than dried flower extract obtained in the same condition in scavenging ABTS radicals. Our data are in agreement also with those reported by Stoilova et al. [40] for commercial elder flower extract in which an IC_50_ value of 0.152 μg/mL was found in the DPPH test. Moreover, *S. nigra* floral extracts exert a greater radical scavenging activity when compared to rutin since it caused 97.70% of DPPH inhibition at 10 μg/mL concentration, while 40 μg/mL of rutin are necessary to ensure DPPH radical inhibition of 77.47%.

### 3.4. Tyrosinase Inhibitory Activity

The search for natural and safe enzyme inhibitors to overcome skin problems is amongst the most investigated matters. These compounds are able to reduce the production of melanin. Melanin is important in protecting skin. However, the accumulation of anomalous quantities of melanin, induced by several factors including UV exposure and drugs, produces more pigmented patches that might become aesthetic problems. Tyrosinase is a key enzyme in the melanogenesis process. In fact, melanogenesis starts with the oxidation of L-tyrosine to dopaquinone by tyrosinase. Despite numerous studies, the actually used inhibitors of tyrosinase still show insufficient activity, low stability, or toxicity. Hence, the search for new tyrosinase inhibitors is very active [5]. Herein, *S. nigra* extracts were investigated for their potential inhibitory activity against mushroom tyrosinase. Data are reported in Table 5.

Generally, flowers are more active than leaves. The most interesting results are obtained by using methanol as a solvent of flower extraction with IC_50_ values of 53.9 and 62.5 μg/mL for retentate fraction and crude extract, respectively. Both extracts are characterized by the presence of rutin (quercetin-3-*O*-rutinoside), quercetin, protocateuchic acid, 3,5-dicaffeoylquinic acid, and kaempferol as the main constituents. Tyrosinase was competitively inactivated by rutin with an IC_50_ value of 6.8 mM and a Ki of 1.10 mM [41]. Rutin competed with L-DOPA in the active site pocket of the enzyme due to the property of copper chelating. A strong copper chelating effect of this flavonoid may be related to the presence of hydroxyl groups. Fan et al. [42] demonstrated that quercetin is able to inhibit both the mono- and di-phenolase activity of tyrosinase, and to inhibit the dopaquinone formation in a reversible competitive manner (IC_50_ of 3.08 × 10^−5^ mol/L). Studies of molecular docking demonstrated the ability of quercetin to chelate a copper with the 3’,4’-dihydroxy groups in the active site of tyrosinase. The chelation may prevent the entrance of substrate with inhibition of the catalytic activity of the enzyme.

## 4. Conclusions

In this work, flower and leaf extracts of *S. nigra* were concentrated using a novel solvent-resistant LMP ECTFE NF membrane in order to concentrate bioactive phytochemicals. Extracts prepared from flowers contained more abundant phenolic compounds than those prepared from leaves and showed the most interesting bioactivities. In particular, the retentate fraction of flowers extracted by methanol exhibited the major tyrosinase inhibitory activity and a promising antioxidant potential. The results obtained in this work suggest that *S. nigra*, particularly its flowers, may be an important dietary source of natural antioxidant and tyrosinase inhibitory agents.

## Figures and Tables

**Table 1:** Properties of the LMP ECTFE N2 membrane (the relative standard error is less than 5% in all cases).

Contact Angle (°)		Mechanical Tests			Porosity (%)	Pore Size Measurements			
Top side	Bottom side	EMod (N/mm^2^)	Tensile strength (N/mm^2^)	eBreak (%)		Bubble point (bar)	Largest pore size (nm)	Mean flow pore diameter (nm)	Diameter at maximum pore size distribution (nm)
105	114	370	17.1	155.4	42.3	2.43	180.0	10.0	10.0

**Table 2:** HPLC profile of selected phenolic acids and flavonoids of *S. nigra* floral extracts.

mg/100 g ex	Ethanol Extracts		Methanol Extracts	
	Crude Extract	Retentate Fraction	Crude Extract	Retentate Fraction
Astragalin	30.1 ± 1.8	37.2 ± 2.1	33.1 ± 1.2	38.4 ± 1.6
Caffeic acid	5.9 ± 0.8	n.d.	8.6 ± 1.8	n.d.
Chlorogenic acid	318.6 ± 4.1	25.7 ± 1.5	460.9 ± 7.5	77.8 ± 1.6
3,5-Dicaffeoylquinic acid	106.1 ± 3.6	140.4 ± 15.5	320.1 ± 13.5	385.7 ± 12.4
Ferulic acid	15.1 ± 0.7	17.0 ± 0.3	28.1 ± 0.5	32.2 ± 0.3
Isoquercetin	57.5 ± 2.7	80.8 ± 5.3	75.9 ± 2.8	93.8 ± 5.3
Kaempferol	237.5 ± 2.9	314.8 ± 7.4	248.6 ± 2.2	394.8 ± 8.6
Myricetin	17.3 ± 2.5	22.6 ± 1.2	17.0 ± 0.6	22.5 ± 1.1
Neochlorogenic acid	112.1 ± 5.0	110.1 ± 3.0	147.5 ± 2.2	137.1 ± 3.0
*p*-Coumaric acid	4.5 ± 0.7	n.d.	4.6 ± 0.5	n.d.
Protocateuchic acid	425.2 ± 11.0	438.6 ± 13.0	433.6 ± 10.0	465.8 ± 13.0
Quercetin	145.5 ± 5.3	254.1 ± 3.7	157.5 ± 4.1	261.1 ± 2.8
Rosmarinic acid	5.5 ± 1.0	9.9 ± 0.5	8.9 ± 0.7	14.7 ± 0.5
Rutin	573.5 ± 5.0	615.9 ± 9.8	611.2 ± 7.2	657.8 ± 8.1

Data are reported as mean ± standard deviation (*n* = 3). n.d.: not detected.

**Table 3:** HPLC profile of selected phenolic acids and flavonoids of *S. nigra* leaf extracts.

mg/100 g ex	Ethanol Extracts		Methanol Extracts	
	Crude Extract	Retentate Fraction	Crude Extract	Retentate Fraction
Astragalin	5.8 ± 1.1	7.2 ± 1.4	5.0 ± 1.8	6.0 ± 1.2
Caffeic acid	2.6 ± 0.6	n.d.	18.8 ± 2.2	n.d.
Chlorogenic acid	52.1 ± 2.3	4.6 ± 1.0	28.6 ± 1.5	2.4 ± 0.8
3,5-Dicaffeoylquinic acid	33.4 ± 4.3	13.1 ± 3.1	62.6 ± 10.5	20.8 ± 1.5
Isoquercetin	49.4 ± 3.2	33.4 ± 3.3	30.2 ± 0.3	20.4 ± 1.4
Kaempferol	37.5 ± 2.4	42.1 ± 1.5	42.6 ± 3.3	38.1 ± 2.7
Myricetin	1.3 ± 0.08	n.d.	1.1 ± 0.06	n.d.
Neochlorogenic acid	65.1 ± 1.5	68.2 ± 1.4	45.6 ± 2.2	45.0 ± 1.9
*p*-Coumaric acid	0.6 ± 0.07	n.d.	0.8 ± 0.05	n.d.
Quercetin	70.3 ± 3.2	160.8 ± 3.5	78.3 ± 2.2	176.5 ± 6.5
Rutin	37.0 ± 1.5	45.7 ± 2.6	44.1 ± 1.5	50.8 ± 2.7

Data are reported as mean ± standard deviation (*n* = 3). n.d.: not detected.

**Table 4:** In vitro antioxidant activity of *S. nigra* leaf and floral extracts.

*S. nigra*	Extraction Solvent	DPPH Test (IC_50_ μg/mL)	ABTS Test (IC_50_ μg/mL)	FRAP Test (μM Fe(II)/g)^a^	RACI
*Leaves*					
Crude extract	methanol	41.3 ± 2.6**	66.0 ± 4.0**	84.4 ± 3.4**	−0.52
	ethanol	42.1 ± 3.1**	80.3 ± 3.3**	102.7 ± 5.9**	0.13
Retentate fraction	methanol	39.2 ± 3.4**	64.3 ± 3.6**	90.1 ± 4.8**	−0.51
	ethanol	40.6 ± 3.8**	78.5 ± 3.2**	110.9 ± 5.3**	0.21
*Flowers*					
Crude extract	methanol	52.4 ± 2.9**	48.2 ± 3.1**	102.6 ± 4.6**	0.03
	ethanol	50.0 ± 3.0**	73.5 ± 4.0**	101.4 ± 5.3**	0.31
Retentate fraction	methanol	48.3 ± 3.8**	46.4 ± 3.5**	109.5 ± 5.7**	−0.01
	ethanol	48.6 ± 3.7**	72.1 ± 5.1**	107.3 ± 4.5**	0.36
Positive control					
Ascorbic acid		5.0 ± 0.8	1.0 ± 0.03		
BHT				63.2 ± 4.5	

Data are given as media ± S.D. (n = 3). Differences within and between groups were evaluated by one-way analysis of variance test *** P < 0.0001 followed by a multicomparison Dunnett’s test: ** P < 0.01 compared with the positive controls. ^a^ Samples tested at the concentration of 2.5 mg/mL.

**Table 5:** In vitro tyrosinase inhibitory activity of *S. nigra* leaf and flower extracts.

*Leaves*	Extraction Solvent	IC_50_ (μg/mL)
Crude extract	methanol	204.5 ± 4.8**
	ethanol	298.4 ± 5.3**
Retentate fraction	methanol	189.3 ± 3.5**
	ethanol	212.7 ± 4.1**
*Flowers*		
Crude extract	methanol	62.5 ± 1.2**
	ethanol	188.2 ± 4.4**
Retentate fraction	methanol	53.9 ± 1.8**
	ethanol	134.7 ± 3.5**
Positive control		
Kojic acid		10.8 ± 0.7

Data are given as media ± S.D. (n = 3). Differences within and between groups were evaluated by one-way analysis of variance test *** P < 0.0001 followed by a multicomparison Dunnett’s test: ** P < 0.01 compared with the positive control.

## References

[1] Charlebois D., Byers P.L., Finn C.E., Thomas A.L., Janick J. (2010). Elderberry: Botany, Horticulture, Potential, in Horticultural Reviews.

[2] Mikulic-Petkovsek M., Samoticha J., Eler K., Stampar F., Veberic R. (2015). Traditional elderflower beverages: A rich source of phenolic compounds with high antioxidant activity. J. Agric. Food Chem..

[3] Uncini Manganelli R.E., Zaccaro L., Tomei P.E. (2005). Antiviral activity in vitro of *Urtica dioica* L., *Parietaria diffusa* M. et K. and *Sambucus nigra* L.. J. Ethnopharmacol..

[4] Xu D.P., Li Y., Meng X., Zhou T., Zhou Y., Zheng J., Zhang J.J., Li H.B. (2017). Natural antioxidants in foods and medicinal plants: Extraction, assessment and resources. Int. J. Mol. Sci..

[5] Sahin S., Samli R., Birteksöz Tan A.S., Barba F.J., Chemat F., Cravotto G., Lorenzo J.M. (2018). Solvent-free microwave-assisted extraction of polyphenols from olive tree leaves: Antioxidant and antimicrobial properties. Molecules.

[6] Bonesi M., Xiao J., Tundis R., Aiello F., Sicari V., Loizzo M.R. (2018). Advances in the tyrosinase inhibitors from plant source. Curr. Med. Chem..

[7] Akin O., Temelli F., Köseoğlu S. (2012). Membrane applications in functional foods and nutraceuticals. Crit. Rev. Food Sci. Nutr..

[8] Castro-Munoz R., Conidi C., Cassano A. (2018). Membrane-based technologies for meeting the recovery of biologically active compounds from foods and their by-products. Crit. Rev. Food Sci. Nutr..

[9] Tundis R., Loizzo M.R., Bonesi M., Sicari V., Ursino C., Manfredi I., Conidi C., Figoli A., Cassano A. (2018). Concentration of bioactive compounds from elderberry (*Sambucus nigra* L.) juice by nanofiltration membranes. Plant Foods Human Nutr..

[10] Marchetti M.F., Solomon J., Szekely G., Livingston A.G. (2014). Molecular separation with organic solvent nanofiltration: A critical review. Chem. Rev..

[11] Nagy E., Nagy E. (2012). Nanofiltration. Basic Equations of Mass Transport Through a Membrane Layer.

[12] Buonomenna M.G., Bae J. (2015). Organic solvent nanofiltration in pharmaceutical industry. Sep. Purif. Rev..

[13] Machado D.R., Hasson D., Semiat R. (1999). Effect of solvent properties on permeate flow through nanofiltration membranes. Part I: Investigation of parameters affecting solvent flux. J. Membr. Sci..

[14] Van der Bruggen B., Geens J., Norman N.L., Anthony G., Fane W.S., Winston Ho T.M. (2008). Nanofiltration in Advanced Membrane Technology and Applications.

[15] Mohammad A.W., Teowa Y.H., Ang W.L., Chung Y.T., Oatley-Radcliffe D.L., Hilal N. (2015). Nanofiltration membranes review: Recent advances and future prospects. Desalination.

[16] Müller H.-J. (2006). A new solvent resistant membrane based on ECTFE. Desalination.

[17] https://www.sartorius.de/mediafile/Manual_Minisart_Chemical_Compatibility_SL-6190-e.pdf..

[18] https://www.sterlitech.com/pub/media/pdf_resources/Chemical_Compatibility.pdf..

[19] Liu F., Hashim N.A., Liu Y., Abed M.R.M., Li K. (2011). Progress in the production and modification of PVDF membranes. J. Membr. Sci..

[20] Amirilargani M., Sadrzadeh M., Sudhölter E.J.R., de Smet L.C.P.M. (2016). Surface modification methods of organic solvent nanofiltration membranes. Chem. Eng. J..

[21] Cui Z., Drioli E., Lee Y.M. (2014). Recent progress in fluoropolymers for membranes. Prog. Polym. Sci..

[22] Ursino C., Simone S., Donato L., Santoro S., De Santo M.P., Drioli E., Di Nicolò E., Figoli A. (2016). ECTFE membranes produced by non-toxic diluents for organic solvent filtration separation. RSC Adv..

[23] Simone S., Figoli A., Santoro S., Galiano F., Alfadul S.M., Al-Harbi A., Omar Drioli E. (2012). Preparation and characterization of ECTFE solvent resistant membranes and their application in pervaporation of toluene/water mixtures. Sep. Purif. Technol..

[24] Falbo F., Santoro S., Galiano F., Simone S., Davoli M., Drioli E., Figoli A. (2016). Organic/organic mixture separation by using novel ECTFE polymeric pervaporation membranes. Polymer.

[25] Drioli E., Santoro S., Simone S., Barbieri G., Brunetti A., Macedonio F., Figoli A. (2014). ECTFE membrane preparation for recovery of humidified gas streams using membrane condenser. Reactive Funct. Polym..

[26] Pan J., Xiao C., Huang Q., Liu H., Hu J. (2015). ECTFE porous membranes with conveniently controlled microstructures for vacuum membrane distillation. J. Mater. Chem. A.

[27] Yao N., Chau J., Elele E., Khusid B., Sirkara K.K., Dehn D. (2017). Characterization of microporous ECTFE membrane after exposure to different liquid media and radiation. J. Membr. Sci..

[28] Loizzo M.R., Tundis R., Chandrika U.G., Abeysekera A.M., Menichini F., Frega N.G. (2010). Antioxidant and antibacterial activities on foodborne pathogens of *Artocarpus heterophyllus* Lam. (*Moraceae*) leaves extracts. J. Food Sci..

[29] Sun T., Tanumihardjo S.A. (2007). An Integrated approach to evaluate food antioxidant capacity. J. Food Sci..

[30] Tundis R., Bonesi M., Pugliese A., Nadjafi F., Menichini F., Loizzo M.R. (2015). Tyrosinase, acetyl- and butyryl-cholinesterase inhibitory activity of *Stachys lavandulifolia* Vahl (*Lamiaceae*) and its major constituents. Rec. Nat. Prod..

[31] Dai J., Mumper R.J. (2010). Plant phenolics: Extraction, analysis and their antioxidant and anticancer properties. Molecules.

[32] Turkmen N., Sari F., Velioglu Y.S. (2006). Effects of extraction solvents on concentration and antioxidant activity of black and black mate tea polyphenols determined by ferrous tartrate and Folin-Ciocalteu methods. Food Chem..

[33] Christensen L.P., Kaack K., Fretté X.C. (2008). Selection of elderberry (*Sambucus nigra* L.) genotypes best suited for the preparation of elderflower extracts rich in flavonoids and phenolic acids. Eur Food Res. Technol..

[34] Dawidowicz A.L., Wianowska D., Baraniak B. (2006). The antioxidant properties of alcoholic extracts from *Sambucus nigra* L. (antioxidant properties of extracts). LWT Food Sci. Technol..

[35] Sidor A., Gramza-Michałowska A. (2015). Advanced research on the antioxidant and health benefit of elderberry (*Sambucus nigra*) in food—A review. J. Funct. Foods.

[36] Thomas A.L., Byers P.L., Finn C.E., Chen Y.C., Rottinghaus G.E., Malone A.M., Applequist W.L. (2008). Occurrence of rutin and chlorogenic acid in elderberry leaf, flower and stem in response to genotype. environment, and season. Acta Horticulture.

[37] Aboul-Enein A.M., El-Baz F.K., El-Baroty G.S., Youssef A.M., Abd-El-Baky H.H. (2003). Antioxidant activity of algal extracts on lipid peroxidation. J. Med. Sci..

[38] Viapiana A., Wesolowski M. (2017). The phenolic contents and antioxidant activities of infusions of *Sambucus nigra* L.. Plant Foods Hum. Nutr..

[39] Młynarczyk K., Walkowiak-Tomczak D. (2017). Bioactive properties of elderflowers (*Sambucus nigra* L.). World Sci. News.

[40] Stoilova I., Wilker M., Stoyanova A., Krastanov A., Stanchev V. (2007). Antioxidant activity of extract from elder flower (*Sambucus nigra* L.). Herba Polonica.

[41] Si Y.X., Yin S.J., Oh S., Wang Z.J., Ye S., Yan L., Yang J.M., Park Y.D., Lee J., Qian G.Y. (2012). An integrated study of tyrosinase inhibition by rutin: Progress using a computational simulation. J. Biomol. Struct. Dyn..

[42] Fan M., Zhang G., Hu X., Xu X., Gong D. (2017). Quercetin as a tyrosinase inhibitor: Inhibitory activity, conformational change and mechanism. Food Res. Int..

